# Kinematic Strategies of Dragonfly Free-Fall Recovery from an Asymmetric Body Roll

**DOI:** 10.3390/insects17050529

**Published:** 2026-05-21

**Authors:** Lingyun Shao, Heyu Li, Jiahao Zhang, Luyao Wang

**Affiliations:** 1 Institute of Plant Protection, Liaoning Academy of Agricultural Sciences, Shenyang 110161, China; lingyunshao@126.com; 2 College of Sciences, Northeastern University, Shenyang 110819, China; liheyu@mails.neu.edu.cn (H.L.); zhangjh10@mails.neu.edu.cn (J.Z.); 3 Key Laboratory of Structural Dynamics of Liaoning Province, College of Sciences, Northeastern University, Shenyang 110819, China

**Keywords:** dragonflies, free-fall recovery, asymmetric roll, kinematics, high-speed videography

## Abstract

Insect aerial righting is a critical locomotor response to flight perturbations. However, the kinematic strategy of dragonfly (*Pantala flavescens*) free-fall recovery under asymmetric perturbations remains unclear. In this study, free-fall experiments were conducted using single-wing release to induce an initial asymmetric body roll, and three-dimensional kinematics were reconstructed using high-speed videography. The results show that the free-fall recovery process was divided into two phases based on body velocity: a free-fall phase with increasing downward velocity and a recovery phase marked by the cessation of this trend and a rise in horizontal velocity. During the free-fall phase, thorax roll developed and then attenuated, accompanied by relative abdominal and wing posture variations, without sustained wing flapping. Near the onset of recovery, hindwings initiated the downstroke earlier than forewings, forming a hindwing-leading flapping pattern. These findings indicate that dragonflies employ a phased kinematic strategy during asymmetric free-fall recovery, involving early body and wing posture adjustments followed by asymmetric wing flapping during the transition from gravity-dominated descent to maneuvering flight.

## Simple Summary

Dragonflies often lose their balance in the air due to sudden gusts of wind or other disturbances. This study explored how dragonflies regain stable flight after an unexpected fall under asymmetric conditions. In the experiments, an asymmetric fall was induced by holding and releasing a single wing, and the body and wing movements were recorded and analyzed. The process was divided into two stages based on body motion. In the first stage, the dragonfly mainly fell downward with increasing speed. During this stage, the body rolled, and the abdomen and wings changed posture, but the wings did not flap continuously. In the second stage, the downward speed stopped increasing and horizontal movement became stronger. Near this transition, the dragonfly began flapping its wings, with the hind wings moving before the front wings. These results show that dragonflies recover from asymmetric falling through body and wing posture adjustments, followed by uneven wing flapping. This study helps us better understand how insects regain flight after disturbances in nature.

## 1. Introduction

Over millions of years of evolution, flying insects have developed sophisticated kinematic strategies to interact with aerial environments, enabling agile maneuvers and effective responses to perturbations [1,2,3,4]. The remarkable flight performance of insects arises from specialized morphological architectures [5,6], flight dynamics involving coordinated body and wing motions [7,8,9], and the utilization of aerodynamics [10,11,12,13,14]. Investigating their kinematic responses is therefore essential for understanding their complex aerial behaviors.

Dragonflies serve as an excellent model system for studying insect flight kinematics [15,16,17]. Unlike dipterans, which possess a single pair of wings, dragonflies feature two pairs of independently controlled wings [18,19,20,21] and a slender, multi-segmented abdomen [22]. The mutual independence of these wings allows for adjustments in phase relationships and stroke trajectories between the forewings and hindwings, thereby modulating the generation of transient aerodynamic forces [21,23,24,25]. Furthermore, research on various insect species indicates that the abdomen can function as an inertial actuator, where rapid flexion shifts the center of mass or alters the moment of inertia to assist in stabilization [1,26,27,28]. This anatomical arrangement permits fine modulation of forewing-hindwing coordination and body posture, supporting a wide repertoire of flight behaviors [25,29,30,31,32,33]. Consequently, dragonflies provide a suitable system for investigating multi-body kinematic coordination during challenging aerial maneuvers.

In their natural habitats, flying insects frequently encounter aerial perturbations that can destabilize flight [34,35,36,37,38,39]. When such disturbances result in free fall, the ability to rapidly restore body orientation before ground contact is critical for survival. The aerial righting reflex thus represents a key survival behavior within the insect locomotor repertoire [26,29,40,41]. This reflex is mediated by the integration of multiple sensory modalities, including visual feedback, mechanosensory input from gyroscopic organs (halteres), tarsal contact, and antennal cues [21,29,40]. While the righting maneuvers triggered by these sensory inputs have been extensively documented in dipterans and other species, the specific kinematic responses in dragonflies, a haltere-free system, during free fall remain less explored. Recent studies have begun to examine the righting reflex in dragonflies, focusing on the sensory inputs and the resulting kinematic responses. Fabian et al. identified a passive mechanism termed “Dragondrop,” in which a stereotyped wing posture combined with abdominal movements generates a pitch-up rotational tendency during falling [26]. This mechanism contributes to attitude recovery during aerial righting. Wang et al. demonstrated that visual feedback triggers active asymmetric wing pitching through the modulation of wing-base muscles, inducing a 180° body roll to recover from upside-down falling within approximately 200 ms [29]. Collectively, these findings establish that both passive aerodynamic mechanisms and active kinematic modulations serve as effective strategies for attitude recovery in dragonflies.

Although these studies have advanced our understanding of the righting reflex, the experimental paradigms employed symmetric pitch-drops [26,29]. Symmetric perturbations primarily engage longitudinal flight dynamics. However, natural disturbances often impose asymmetric loads that lead to coupled rotations across multiple axes. The recovery from such states requires more complex coordination of bilateral wing kinematics and multi-body postures [34,35,42,43]. How dragonflies coordinate their multi-segmented body and four independent wings to recover from an initial asymmetric, gravity-dominated descent therefore remains insufficiently characterized.

To address this gap, we conducted free-fall experiments on dragonflies using a single-wing release method to induce an initial asymmetric body roll. The resulting three-dimensional (3D) kinematics of the multi-segmented body and independent wings were captured using high-speed videography. By analyzing the time-resolved evolution of body posture and wing kinematics, the present study aims to elucidate the kinematic strategies employed during free-fall recovery under asymmetric perturbations.

## 2. Materials and Methods

### 2.1. Experimental Subjects and Free-Fall Experimental Setup

Free-fall experiments were conducted using wild-caught adult dragonflies (*Pantala flavescens*). Specimens were collected in Shenyang, Liaoning Province, China, in September 2023. To preserve the physiological integrity and minimize potential changes in kinematic responses, all dragonflies were tested within 24 h of capture. All procedures adhered to the 3Rs principles (Replacement, Reduction, Refinement) and complied with institutional and national guidelines for the ethical treatment of animals. As the study involved invertebrates, formal ethical approval was not required under the policies of Northeastern University (China); nevertheless, every effort was made to minimize handling time and potential distress. Morphological parameters of the representative experimental dragonfly are provided in Table 1.

**Figure 1:**
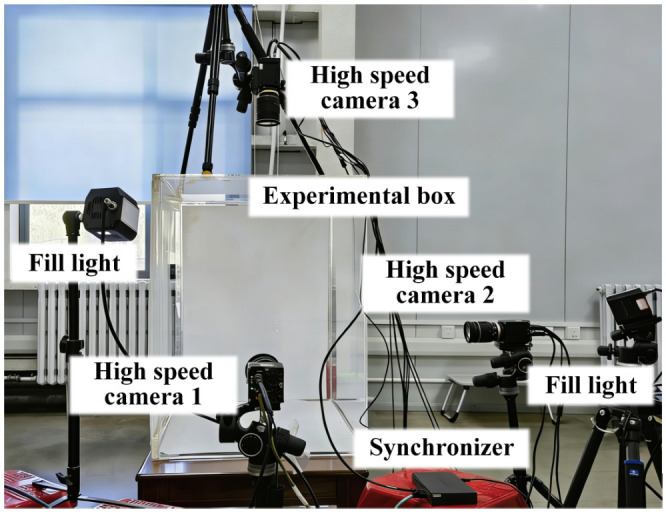
Experimental setup of the high-speed videography system for recording the free-fall recovery events of dragonflies. The system consists of three synchronized high-speed cameras, two fill lights, and a transparent experimental box.

The kinematics of dragonflies during the free-fall recovery events were captured using a synchronized high-speed videography system (Figure 1). Experiments were performed inside a transparent acrylic box with internal dimensions of 800 × 800 × 600 mm^3^. This volume was chosen to minimize potential aerodynamic wall and ground effects during dragonfly free-fall recovery events. Three high-speed cameras (Revealer M230M/C, HF Agile Device Co., Ltd., Hefei, Anhui, China) operating at 3000 frames per second (fps) with a spatial resolution of 1920 × 1080 pixels were positioned approximately orthogonally to record top, front, and side views of the calibrated flight volume. Fill lights and white diffusion paper on the background walls were used to ensure sufficient illumination and high-contrast silhouettes for subsequent digital tracking.

In each event, the dragonfly was manually held by a single wing near the top of the calibrated field of view and then released, thereby inducing an asymmetric initial body posture and rolling motion. Care was taken to ensure that the release process introduced minimal additional perturbation beyond the intended single-wing asymmetry. The posture and angular motion after release were not prescribed; therefore, event-dependent initial conditions were expected. Upon release, the multi-camera system was triggered simultaneously via a central synchronizer to record the entire free-fall recovery event.

### 2.2. Three-Dimensional Calibration and Kinematic Data Extraction

**Figure 2:**
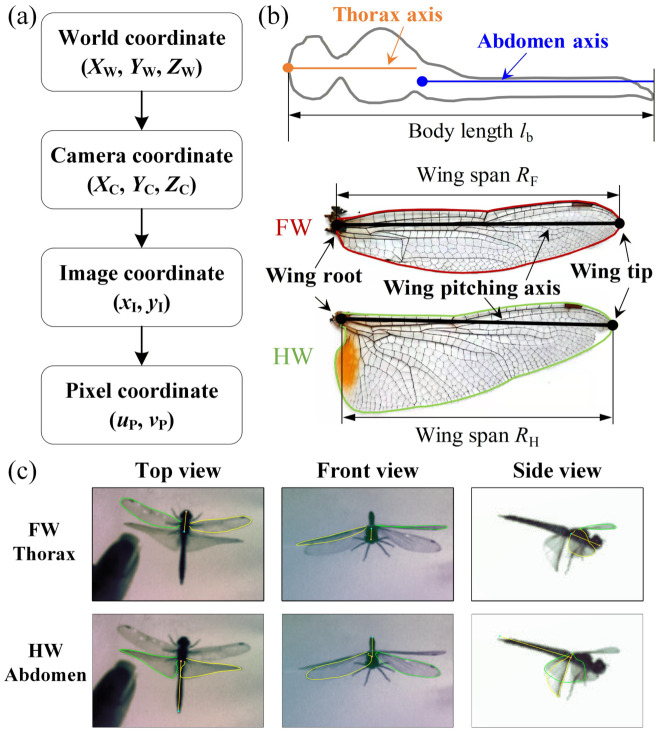
Three-dimensional calibration and kinematic data extraction methodology. (**a**) Schematic of the coordinate transformation pipeline from world coordinates to camera, image, and pixel coordinates. (**b**) Morphological definitions of a representative dragonfly (*Pantala flavescens*) specimen, showing the independent longitudinal axes of the thorax and abdomen, as well as the extracted wing contours, pitching axes, and spans for forewing (FW) and hindwing (HW). The HW exhibits a characteristic broadened basal area and a yellowish pigmented patch. (**c**) Representative high-speed video frames demonstrating the visual matching tracking process. The top row shows the fitted model for the thorax and FWs; the bottom row shows independent tracking for the abdomen and HWs in top, front, and side views.

To reconstruct the 3D positions of the dragonfly during the free-fall recovery events, the multi-camera imaging system was calibrated prior to the experiments (Figure 2a). The spatial mapping from 3D world coordinates (*X*_W_, *Y*_W_, *Z*_W_) to two-dimensional (2D) pixel coordinates (*u*_P_, *v*_P_) was performed using the pinhole camera model combined with Zhang’s checkerboard calibration method [44]. The transformation, incorporating intrinsic and extrinsic camera parameters, is expressed as follows:

(1) $$Z_{C}\left[\begin{array}{l} u_{P}\\ v_{P}\\ 1 \end{array}\right]=\left[\begin{array}{ccc} 1/dx_{I} & 0 & u_{P0}\\ 0 & 1/dy_{I} & v_{P0}\\ 0 & 0 & 1 \end{array}\right]\left[\begin{array}{ccc} 1+kr_{I}^{2} & 0 & 0\\ 0 & 1+kr_{I}^{2} & 0\\ 0 & 0 & 1 \end{array}\right]\left[\begin{array}{cccc} f_{e} & 0 & 0 & 0\\ 0 & f_{e} & 0 & 0\\ 0 & 0 & 1 & 0 \end{array}\right]\left[\begin{array}{cc} R & T\\ 0 & 0 \end{array}\right]\left[\begin{array}{c} X_{W}\\ Y_{W}\\ Z_{W}\\ 1 \end{array}\right].$$

Here, *Z*_C_, *k*, and *f*_e_ represent the camera depth, radial distortion parameter, and effective focal length, respectively. The physical pixel dimensions of the camera are denoted by d*x*_I_ and d*y*_I_, while (*u*_P0_, *v*_P0_) specify the pixel coordinates of the image center. The variable *r*_I_ is the radial distance from the projected point to the optical axis, calculated as follows:

(2) $$r_{I}=\sqrt{x_{I}^{2}+y_{I}^{2}}.$$

Finally, **R** and **T** are the rigid-body rotation matrix and translation vector, representing the transformation from the 3D world coordinate system (*X*_W_, *Y*_W_, *Z*_W_) to the camera coordinate system (*X*_C_, *Y*_C_, *Z*_C_).

To determine the calibration parameters, a black-and-white checkerboard [44] (8 × 11 squares, 45 mm grid size) was placed at multiple positions and orientations within the flight volume. Calibration images were captured for each camera following standard procedures. The mean reprojection error for individual cameras was below 0.01 pixels, and the multi-camera correlation error was below 0.1 pixels, indicating high reconstruction accuracy for three-dimensional kinematic analysis.

Prior to kinematic tracking, morphological templates were constructed for the dragonfly specimen from high-resolution digital photographs (Figure 2b). Longitudinal axes were defined for the thorax and abdomen. Wing contours, wing pitching axes, and wing spans (*R*_F_ and *R*_H_) for both the forewing (FW) and hindwing (HW) were also extracted to serve as morphological references.

Time-resolved kinematic data were extracted from the synchronized high-speed videos using a custom MATLAB R2024b program based on a 3D-to-2D projection and visual matching technique [14,45,46,47,48]. Briefly, each video sequence was decomposed into individual frames. Given that the camera frame rate (3000 fps) greatly exceeded the dragonfly wingbeat frequency, frames were subsampled during data extraction according to the flapping frequency and the temporal resolution required for kinematic analysis. The 3D morphological templates of the thorax, abdomen, FWs, and HWs were then projected onto the image planes of all three camera views. The spatial position and rotational angles of the model were adjusted until the projected outlines achieved precise alignment with the actual insect silhouettes in all orthogonal views simultaneously (Figure 2c). The resulting 3D coordinates and orientation angles at each frame were recorded as the instantaneous kinematics. This procedure allowed the thorax, abdomen, and four wings to be reconstructed separately. The detailed principles and validation of this visual matching approach have been described previously [46].

To reduce high-frequency noise introduced by the digitization process, the raw kinematic time series were smoothed using a Savitzky–Golay filter. Because body and wing motions contain different frequency components, separate filtering parameters were applied. For translational and rotational displacements of the thorax and abdomen, a polynomial order of 3 and a frame window of 11 were used. For wing flapping, deviation, and pitching angles, a polynomial order of 3 and a frame window of 7 were applied to better preserve high-frequency flapping features. All velocities were subsequently calculated from the smoothed datasets.

### 2.3. Coordinate Systems and Kinematics

**Figure 3:**
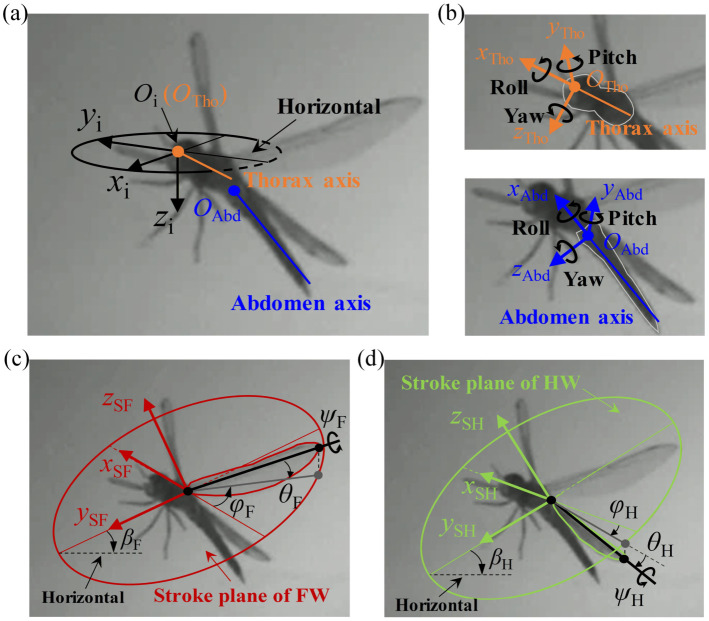
Definitions of coordinate systems and kinematics used to quantify dragonfly flight kinematics during the free-fall recovery events. (**a**) Translating coordinate system (*O*_i_-*x*_i_*y*_i_*z*_i_) describing spatial translation of the body. (**b**) Body-fixed coordinate systems for the thorax (*O*_Tho_-*x*_Tho_*y*_Tho_*z*_Tho_) and abdomen (*O*_Abd_-*x*_Abd_*y*_Abd_*z*_Abd_), with the definitions of rotational Euler angles: yaw, roll, and pitch. (**c**) and (**d**) Stroke-plane coordinate systems *O*_SF_-*x*_SF_*y*_SF_*z*_SF_ for FW and *O*_SH_-*x*_SH_*y*_SH_*z*_SH_ for HW, respectively. Stroke planes are defined by linear regression of wing-tip trajectories. The solid gray lines in panel (**c**) and (**d**) indicate the projection of the wing pitching axis onto the stroke plane. *β*_F_ and *β*_H_ denote the stroke-plane angles relative to the horizontal plane. Wing kinematics are described by three Euler angles: flapping angle (*φ*_F_, *φ*_H_), deviation angle (*θ*_F_, *θ*_H_), and wing pitching angle (*ψ*_F_, *ψ*_H_).

The flight kinematics of the dragonfly free-fall recovery events involve the coupled motions of a multi-segmented body and four independently controlled wings. To quantify these motions, a series of coordinate systems and kinematic variables were defined (Figure 3).

As illustrated in Figure 3a, the translating coordinate system (*O*_i_-*x*_i_*y*_i_*z*_i_) was used to describe spatial translation of the body. Due to the articulated structure of the dragonfly body, independent body-fixed coordinate systems were established for the thorax (*O*_Tho_-*x*_Tho_*y*_Tho_*z*_Tho_) and the abdomen (*O*_Abd_-*x*_Abd_*y*_Abd_*z*_Abd_) (Figure 3b). These coordinate systems include the definitions of rotational Euler angles: yaw (*Ψ*_Tho_, *Ψ*_Abd_), pitch (*Θ*_Tho_, *Θ*_Abd_), and roll (*Φ*_Tho_, *Φ*_Abd_). Additionally, the relative orientation of the abdomen with respect to the thorax was determined by computing the relative rotation matrix from the thorax coordinate system to the abdomen coordinate system. Using the same rotation sequence, the relative Euler angles (relative yaw Δ*Ψ* and relative pitch Δ*Θ*) were extracted. These variables describe abdominal motion relative to the thorax and are independent of the global rotation of the whole body.

To quantify the motions of the FW and HW, individual stroke plane coordinate systems (*O*_SF_-*x*_SF_*y*_SF_*z*_SF_ and *O*_SH_-*x*_SH_*y*_SH_*z*_SH_) were established with their origins fixed at the respective wing roots (Figure 3c,d). The stroke plane was defined by a linear regression plane of the wing-tip trajectory points over the analyzed free-fall recovery sequence. The fitted plane was then used as a common geometric reference for calculating the wing Euler angles in both the free-fall phase and the recovery phase. The inclination of stroke plane relative to the horizontal yielded the stroke-plane angle (*β*_F_ and *β*_H_). Within the stroke plane coordinate systems, the instantaneous spatial orientation of each wing was described by three Euler angles. The flapping angle (*φ*_F_, *φ*_H_) represented the angular position of the wing sweeping parallel to the stroke plane. The deviation angle (*θ*_F_, *θ*_H_) was calculated as the angle between the wing pitching axis and its projection onto the stroke plane (indicated by the solid gray lines in Figure 3c,d). The rotation of the wing surface around its own pitching axis was defined as the wing pitching angle (*ψ*_F_, *ψ*_H_).

The flapping period (*T*) was defined as the time interval between two consecutive onsets of the downstroke. The flapping frequency was calculated as *f* = 1/*T*. The flapping amplitude (*A_φ_*) and pitching amplitude (*A_ψ_*) were defined as the peak-to-peak differences between the maximum and minimum angles within a single flapping cycle:

(3) $$A_{\varphi }=\varphi_{\max}-\varphi_{\min}\;\mathrm{and}\;A_{\psi }=\psi_{\max}-\psi_{\min}.$$

The mean flapping angle ($\bar{\varphi }$) and mean pitching angle ($\bar{\psi }$) were defined as the mean of the respective maximum and minimum values:

(4) $$\bar{\varphi }=\frac{\varphi_{\max}+\varphi_{\min}}{2}\;\mathrm{and}\;\bar{\psi }=\frac{\psi_{\max}+\psi_{\min}}{2}.$$

The phase difference (*γ*) between the FW and HW was defined as the time delay (Δ*t*_d_) between their respective downstroke onsets (*t*_d,F_ and *t*_d,H_), divided by the FW flapping period (*T*_F_):

(5) $$\gamma =\Delta t_{d}/T_{F}\times 360{}^{\circ}=(t_{d,F}-t_{d,H})/T_{F}\times 360{}^{\circ}.$$

### 2.4. Free-Fall Recovery Event and Phase Definition

Three free-fall recovery events by single-wing release were quantitatively reconstructed and denoted as Event 1, Event 2, and Event 3. These events were analyzed because continuous three-dimensional kinematic time series of the main body posture and wing motion could be obtained over the interval from release to recovery. Because of the limited number of successfully reconstructed free-fall recovery events, the present study focuses on event-wise kinematic observations rather than population-level statistical inference. The objective is to identify consistent kinematic features associated with the individual free-fall recovery events.

The release moment was defined as *t* = 0. Two characteristic time points, *t*_Roll_ and *t*_Rec_, were identified from the smoothed kinematic time series. The time *t*_Roll_ was defined as the turning point of the thorax roll angle *Φ*_Tho_, where the increase in *Φ*_Tho_, ceased and the roll angle began to decrease. Thus, *t*_Roll_ indicates the onset of thorax roll attenuation after the release-induced motion. The time *t*_Rec_ was defined as the onset of recovery, marking the transition from the free-fall phase to the recovery phase. This transition was identified by the cessation of the increase in the magnitude of downward vertical velocity, together with the rise of horizontal velocity components. Based on *t*_Rec_, the analyzed sequence was divided into the free-fall phase (*t* < *t*_Rec_) and the recovery phase (*t* ≥ *t*_Rec_).

## 3. Results and Discussion

### 3.1. Translational Kinematics and Phase Division

**Figure 4:**
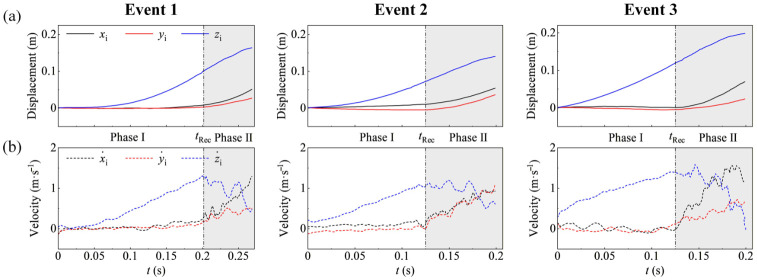
Translational kinematics of dragonflies during all free-fall recovery events. (**a**) Time histories of displacements and (**b**) velocities in the translating coordinate system. The vertical dash-dotted lines indicate the onset of recovery (*t*_Rec_), marking the transition from the free-fall phase to the recovery phase. This transition is characterized by the cessation of the increase in downward vertical velocity (${\dot{z}}_{i}$) and the simultaneous rise in horizontal velocities (${\dot{x}}_{i}$ and ${\dot{y}}_{i}$). The unshaded regions represent the free-fall phase (Phase I), while the gray shaded areas represent the recovery phase (Phase II).

The translational kinematics, including both displacements and velocities, were analyzed to characterize the free-fall recovery events (Figure 4).

As shown in Figure 4, each free-fall recovery event was divided into two phases: the free-fall phase (*t* < *t*_Rec_) and the recovery phase (*t* ≥ *t*_Rec_). At *t*_Rec_, the downward vertical velocity (${\dot{z}}_{i}$) ceased to increase, while the horizontal velocities (${\dot{x}}_{i}$ and ${\dot{y}}_{i}$) began to rise.

During the free-fall phase (*t* < *t*_Rec_), the dragonflies exhibited a gravity-dominated descent. As shown in Figure 4a, the vertical displacement (*z*_i_) increased continuously, while the horizontal displacements (*x*_i_ and *y*_i_) remained close to zero. The corresponding velocities (Figure 4b) showed that the downward vertical velocity (${\dot{z}}_{i}$) generally increased with time, although the linearity varied slightly across all free-fall recovery events. In contrast, the horizontal velocities (${\dot{x}}_{i}$ and ${\dot{y}}_{i}$) fluctuated near zero, indicating a predominantly vertical trajectory.

Following this onset (*t* ≥ *t*_Rec_), the dragonflies transitioned into the recovery phase. The downward vertical velocity (${\dot{z}}_{i}$) ceased to increase in magnitude and then showed either a fluctuating plateau or a decreasing trend (Figure 4b). During this phase, the velocity curves exhibited clear intra-cycle oscillations, corresponding to the cyclic flapping motions of the wings and marking the shift from gravity-dominated descent to maneuvering flight.

### 3.2. Body Attitude and Abdominal Relative Posture

**Figure 5:**
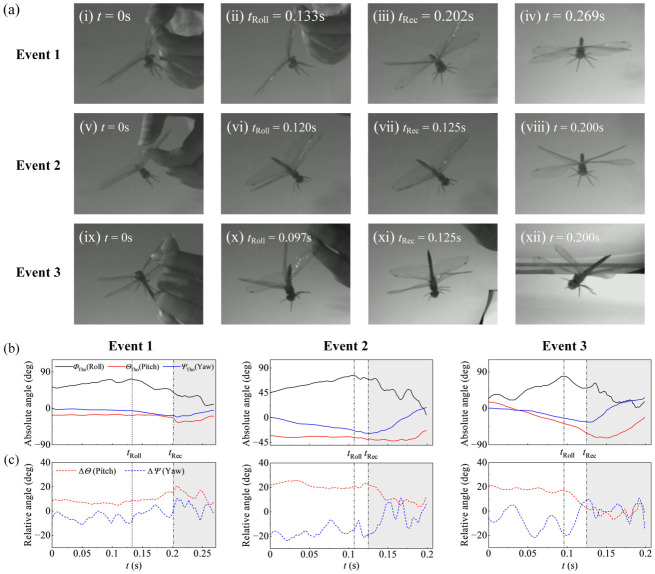
Body posture kinematics of the dragonflies in all free-fall recovery events. (**a**) Representative high-speed video frames at selected time points. (**b**) Time histories of absolute Euler angles of the thorax (*Φ*_Tho_, *Θ*_Tho_, *Ψ*_Tho_). (**c**) Time histories of relative Euler angles of the abdomen with respect to the thorax (Δ*Θ*, Δ*Ψ*), describing thorax–abdomen relative posture. The vertical dotted lines indicate the onset of *Φ*_Tho_ reduction at *t*_Roll_.

The postural changes in the thorax and abdomen for the dragonflies in all free-fall recovery events were quantified in Figure 5. The initial single-wing release induced an asymmetric body posture (Figure 5a).

During the free-fall phase (*t* < *t*_Rec_), the thorax roll angle (*Φ*_Tho_) increased continuously after release, indicating the persistence of the initial rolling motion induced by the asymmetric release condition (Figure 5b). The increase in *Φ*_Tho_ continued until *t*_Roll_, after which the *Φ*_Tho_ began to decrease prior to *t*_Rec_. In the recovery phase (*t* ≥ *t*_Rec_), *Φ*_Tho_ decreased continuously. The thorax pitch (*Θ*_Tho_) and yaw (*Ψ*_Tho_) angles exhibited variations across all free-fall recovery events (Figure 5b).

The relative kinematics of the abdomen with respect to the thorax throughout all free-fall recovery events are shown in Figure 5c. During the free-fall phase (*t* < *t*_Rec_), the abdomen generally remained ventrally deflected relative to the thorax, as indicated by the positive relative pitch angle (Δ*Θ*). The temporal patterns of Δ*Ψ* and Δ*Θ* differed among Events 1–3, indicating trial-to-trial kinematic variability rather than a consistent or stereotyped abdominal motion pattern. Thus, while the abdomen remained mobile relative to the thorax, its motion did not follow a fixed pitch or yaw trajectory across all events.

The continued increase in *Φ*_Tho_ before *t*_Roll_ is consistent with the continuation of the release-induced rolling motion caused by the asymmetric initial condition, because sustained periodic wing flapping was not yet observed during this interval. After *t*_Roll_, *Φ*_Tho_ began to decrease before the onset of the recovery phase, indicating an attenuation of thorax roll during the late free-fall phase. During the same interval, variations in Δ*Θ* and Δ*Ψ* were observed, indicating a temporal correlation between thorax roll dynamics and abdominal posture adjustments. Previous studies have shown that abdominal posture adjustment can shift the center of mass and alter the body’s moment of inertia, thereby influencing rotational motion in insects [1,26,27,28]. Thus, in the present study, the variations in Δ*Θ* and Δ*Ψ* occurred together with the attenuation of thorax roll, which may reflect changes in body configuration and inertial properties. However, because inertial torques were not directly quantified, this interpretation should be regarded as kinematic associations rather than direct dynamic evidence.

### 3.3. Wing Kinematics

**Figure 6:**
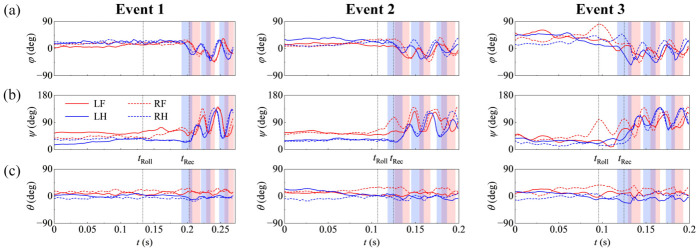
Wing kinematics of the dragonflies in all free-fall recovery events. Time histories of Euler angles for the four wings: (**a**) flapping angle (*φ*), (**b**) pitching angle (*ψ*) and (**c**) deviation angle (*θ*). The solid and dashed lines represent the left (L) and right (R) wings, respectively, while the red and blue colors denote the FWs (F) and HWs (H). In panel (**a**), key kinematic parameters are annotated on the representative wing: stroke amplitude of FW (*A_φ_*_,F_), flapping period of FW (*T*_F_), and the time delay (Δ*t*_d_) between the downstroke onsets of HW (*t*_d,H_) and FW (*t*_d,F_). The shaded regions indicate the downstroke phases for the FWs (red) and HWs (blue), respectively. The purple shaded regions indicate the overlap between the downstroke phases of the FWs and HWs.

Wing kinematics of the dragonflies in all free-fall recovery events are presented in Figure 6. During the free-fall phase (*t* < *t*_Rec_), the wings displayed asymmetric postures without sustained periodic flapping. Prior to *t*_Roll_ (*t* < *t*_Roll_), the variations in the wing kinematics remained small. Specifically, both flapping angles (*φ*) and deviation angles (*θ*) showed only low-amplitude fluctuations, while pitching angles (*ψ*) exhibited gradual changes. During the interval from *t*_Roll_ to *t*_Rec_, *ψ* showed more noticeable changes across all free-fall recovery events. In Event 3 (Figure 6), an additional localized asymmetric variation was observed in the right forewing (RF), with distinct changes in both *φ* and *ψ* near *t*_Roll_. However, this localized kinematic response was not observed in the other free-fall recovery events. The changes in wing posture before *t*_Rec_ indicate that the wing surfaces were not static during the free-fall phase. Instead, the wings underwent posture adjustments before the onset of periodic flapping.

Sustained periodic wing flapping in the dragonfly free-fall recovery events commenced asynchronously around *t*_Rec_ (Figure 6). In all free-fall recovery events, the HWs initiated the first downstroke (*t*_d,H_) prior to the FWs (*t*_d,F_). Specifically, at *t*_Rec_, the increase in the magnitude of downward vertical velocity ceased, and the horizontal velocity components began to rise. This transition occurred when the HWs were near the middle of their initial downstroke, whereas the FWs were close to their downstroke onset (*t*_d,F_). This hindwing-leading pattern is quantified by the phase difference (γ) presented in Table 2. All four wings began the recovery phase with a downstroke (Figure 6a). Subsequently, the flapping angles (*φ*) and pitching angles (*ψ*) developed into continuous, periodic oscillations, although a certain degree of left–right asymmetry persisted across wing pairs (Figure 6). These results indicate that the transition from the free-fall phase to the recovery phase was characterized by asynchronous wing kinematics, in which the HWs preceded the FWs at the onset of recovery (*t*_Rec_).

Table 2 summarizes the kinematic parameters for the four wings of the dragonflies in all free-fall recovery events during the first two cycles of the recovery phase. Here, *T* represents the duration of the flapping cycle. The flapping amplitude (*A_φ_*), mean flapping angle ($\bar{\varphi }$), and pitching amplitude (*A_ψ_*) exhibited considerable inter-event and inter-wing variations, reflecting the unsteady kinematic transitions during the recovery phase. The mean pitching angle ($\bar{\psi }$) exhibited an inter-cycle pattern. Specifically, during Cycle 1, the $\bar{\psi }$ values for HWs were smaller than those for FWs in all free-fall recovery events. By Cycle 2, however, $\bar{\psi }$ of the HWs increased and became comparable to FWs. In addition, the HWs operated at larger stroke-plane angles (*β*) than the FWs.

### 3.4. Phased Kinematic Strategy of Asymmetric Free-Fall Recovery in Dragonflies

The results in Section 3.1, Section 3.2 and Section 3.3 collectively show a two-phase kinematic sequence of dragonflies during the free-fall recovery process, which differs from the rapid righting maneuvers reported in other insect orders. For example, dipterans typically initiate aerial righting within milliseconds of a perturbation, relying on haltere-mediated sensory feedback to generate corrective aerodynamic torques through rapid wing flapping [40,41,49].

In contrast, the present results show that dragonflies experience a free-fall phase without sustained periodic wing flapping. During this period, variations in relative abdominal angles and wing posture occurred in the late free-fall phase, when thorax roll began to attenuate after *t*_Roll_. This posture-dependent kinematic behavior involving abdominal posture adjustment during descent is consistent with observations in some flightless and wingless insects [50,51].

Following the free-fall phase, the motion entered the recovery phase at *t*_Rec_, where asynchronous wing flapping was initiated. At this moment, the HWs had already entered their initial downstroke, whereas the FWs were close to their downstroke onset. Thus, the beginning of the recovery phase was characterized by a hindwing-leading flapping sequence during the transition to maneuvering flight.

Overall, the free-fall recovery process observed in dragonflies involves early body and wing posture adjustments and subsequent asymmetric wing kinematics, providing kinematic evidence at the event level for a phased recovery sequence after asymmetric perturbations. Because aerodynamic forces, aerodynamic moments, pressure distributions, and inertial torques were not directly quantified, the observed abdominal posture adjustment and hindwing-leading flapping pattern should be interpreted as kinematic associations rather than direct dynamic evidence. Future studies with larger sample sizes, repeated trials from identifiable individuals, and aerodynamic or numerical analyses are needed to test the generality of this sequence and clarify the underlying force and moment mechanisms.

## 4. Conclusions

This study characterized the kinematic strategy of dragonfly free-fall recovery following asymmetric perturbations induced by single-wing release. Based on body translational velocity, the free-fall recovery process was divided into two phases. The free-fall phase was characterized by a continuous increase in downward velocity, whereas the recovery phase began when this increase ceased and horizontal velocity components became more pronounced.

Within this velocity-defined framework, the two phases showed distinct kinematic features. During the free-fall phase, thorax roll developed and subsequently attenuated, accompanied by variations in relative abdominal and wing postures, while sustained wing flapping was absent. Near the onset of recovery, wing flapping was initiated asynchronously, with the hindwings starting downstroke earlier than the forewings. This hindwing-leading pattern was observed across all free-fall recovery events.

Overall, these findings indicate that dragonflies recover from asymmetric free fall through a phased kinematic sequence involving body velocity transition, body and wing posture adjustments, and coordinated asymmetric wing flapping. This study provides event-level kinematic evidence for how dragonflies transition from gravity-dominated rolling descent to maneuvering flight under asymmetric perturbations.

## Figures and Tables

**Table 1:** Morphometric parameters of the representative dragonfly.

Parameter		Symbol (Unit)	Value
Body	Length	*l*_b_ (mm)	49.68
	Mass	*m* (mg)	396.52
Forewing (FW)	Span length	*R*_F_ (mm)	44.17
	Area	*S*_F_ (mm^2^)	371.83
	Chord length	*c*_F_ (mm)	8.42
	Radius of the second moment of wing area	*r*_2_F_ (mm)	24.86
Hindwing (HW)	Span length	*R*_H_ (mm)	41.83
	Area	*S*_H_ (mm^2^)	511.37
	Chord length	*c*_H_ (mm)	12.23
	Radius of the second moment of wing area	*r*_2_H_ (mm)	21.06

**Table 2:** Wing kinematic parameters during the first two flapping cycles of the recovery phase in the dragonfly free-fall recovery events.

	Wing	*T* (s)	*A_φ_* (deg)	$\bar{\varphi }$ (deg)	*A_ψ_* (deg)	$\bar{\psi }$ (deg)	*γ* (deg)	*β* (deg)
Cycle 1 of Event 1	LF	0.029	28.75	2.97	64.93	76.24	137.01	38.42
	RF	0.032	40.39	2.42	82.65	92.98		
	LH	0.031	40.98	2.55	50.49	50.01	-	56.78
	RH	0.035	30.56	4.48	39.75	50.62		
Cycle 2 of Event 1	LF	0.027	57.79	−5.44	91.79	89.50	121.86	-
	RF	0.027	56.69	−12.05	95.96	89.74		
	LH	0.028	63.28	−6.45	93.15	82.89	-	-
	RH	0.026	58.76	−3.46	97.91	86.92		
Cycle 1 of Event 2	LF	0.028	39.24	−11.15	68.46	89.77	117.00	40.78
	RF	0.027	49.67	−1.59	76.67	98.93		
	LH	0.028	36.15	6.50	51.35	52.08	-	48.46
	RH	0.027	27.49	13.84	40.43	51.24		
Cycle 2 of Event 2	LF	0.027	34.98	−5.08	47.54	90.22	103.50	-
	RF	0.025	63.20	−1.24	73.08	96.22		
	LH	0.029	54.72	−5.44	80.99	80.14	-	-
	RH	0.027	57.66	0.25	80.53	86.01		
Cycle 1 of Event 3	LF	0.029	38.08	−5.88	49.47	81.77	167.44	39.27
	RF	0.029	58.65	−11.10	90.24	85.26		
	LH	0.034	66.48	−24.39	60.77	68.12	-	52.34
	RH	0.029	79.81	2.54	79.19	65.84		
Cycle 2 of Event 3	LF	0.023	42.30	−5.97	41.56	95.74	118.29	-
	RF	0.023	64.83	−3.44	74.05	98.70		
	LH	0.029	44.66	−11.39	67.53	102.40	-	-
	RH	0.026	60.53	−3.72	79.51	98.02		

## Data Availability

The raw data supporting the conclusions of this article will be made available by the authors on request.
